# Network-based analysis reveals novel gene signatures in peripheral blood of patients with chronic obstructive pulmonary disease

**DOI:** 10.1186/s12931-017-0558-1

**Published:** 2017-04-24

**Authors:** Ma’en Obeidat, Yunlong Nie, Virginia Chen, Casey P. Shannon, Anand Kumar Andiappan, Bernett Lee, Olaf Rotzschke, Peter J. Castaldi, Craig P. Hersh, Nick Fishbane, Raymond T. Ng, Bruce McManus, Bruce E. Miller, Stephen Rennard, Peter D. Paré, Don D. Sin

**Affiliations:** 1 The University of British Columbia Centre for Heart Lung Innovation, St Paul’s Hospital, 1081 Burrard Street, Vancouver, BC V6Z 1Y6 Canada; 2 Prevention of Organ Failure (PROOF) Centre of Excellence, Vancouver, BC Canada; 3 Singapore Immunology Network, 8A Biomedical Grove, Singapore, Singapore; 4 Channing Division of Network Medicine, Brigham and Women’s Hospital, Boston, USA; 5 Division of General Internal Medicine and Primary Care, Brigham and Women’s Hospital and Harvard Medical School, Boston, USA; 6 Pulmonary and Critical Care Division, Brigham and Women’s Hospital and Harvard Medical School, Boston, USA; 7 GlaxoSmithKline, King of Prussia, PA USA; 8 Division of Pulmonary and Critical Care Medicine, University of Nebraska Medical Center, Omaha, NE USA; 9 Clinical Discovery Unit, Early Clinical Development, AstraZeneca, Cambridge, UK; 10 Respiratory Division, Department of Medicine, University of British Columbia, Vancouver, BC Canada

**Keywords:** COPD, FEV_1_, Blood, mRNA, Gene expression, Co-expression, WGCNA, Biomarker, Transcriptome

## Abstract

**Background:**

Chronic obstructive pulmonary disease (COPD) is currently the third leading cause of death and there is a huge unmet clinical need to identify disease biomarkers in peripheral blood. Compared to gene level differential expression approaches to identify gene signatures, network analyses provide a biologically intuitive approach which leverages the co-expression patterns in the transcriptome to identify modules of co-expressed genes.

**Methods:**

A weighted gene co-expression network analysis (WGCNA) was applied to peripheral blood transcriptome from 238 COPD subjects to discover co-expressed gene modules. We then determined the relationship between these modules and forced expiratory volume in 1 s (FEV_1_). In a second, independent cohort of 381 subjects, we determined the preservation of these modules and their relationship with FEV_1_. For those modules that were significantly related to FEV_1_, we determined the biological processes as well as the blood cell-specific gene expression that were over-represented using additional external datasets.

**Results:**

Using WGCNA, we identified 17 modules of co-expressed genes in the discovery cohort. Three of these modules were significantly correlated with FEV_1_ (FDR < 0.1). In the replication cohort, these modules were highly preserved and their FEV_1_ associations were reproducible (*P* < 0.05). Two of the three modules were negatively related to FEV_1_ and were enriched in IL8 and IL10 pathways and correlated with neutrophil-specific gene expression. The positively related module, on the other hand, was enriched in DNA transcription and translation and was strongly correlated to CD4+, CD8+ T cell-specific gene expression.

**Conclusions:**

Network based approaches are promising tools to identify potential biomarkers for COPD.

**Trial registration:**

The ECLIPSE study was funded by GlaxoSmithKline, under ClinicalTrials.gov identifier NCT00292552 and GSK No. SCO104960

**Electronic supplementary material:**

The online version of this article (doi:10.1186/s12931-017-0558-1) contains supplementary material, which is available to authorized users.

## Background

Chronic obstructive pulmonary disease (COPD) is currently the third leading cause of death [1]. The disease is under genetic and environmental control with cigarette smoking being the major modifiable risk factor in the Western world [2]. COPD is characterized by chronic irreversible airflow limitation that is often accompanied by systemic inflammation [3, 4]. The two main morphologic phenotypes of COPD are small airway obstruction and emphysematous destruction and enlargement of airspaces. While the molecular mechanisms underlying the two processes may be different, COPD is diagnosed and assessed using lung function parameters; the most commonly used are the forced expiratory volume in 1 s (FEV_1_) and its ratio with the forced vital capacity (FEV_1_/FVC).

There is a huge unmet clinical need to identify clinically useful biomarkers for COPD [5]. To this end, blood biomarkers would be highly desirable since blood is very accessible. However, the main limitation of blood as a source for biomarker discovery is that its signals may not reflect the disease process in lungs, which are the predominant site of disease in COPD. Recently, a number of studies have evaluated the relationship of gene expression profiles in peripheral blood with COPD endpoints and have demonstrated some signal [6, 7]. One major limitation of using gene expression data for biomarker discovery is the requirement for statistical stringency in determining significant expression changes. However, biologically, this traditional approach lacks intuition since genes are expressed (and function) in clusters or networks rather than as independent entities.

To address this limitation, in this study, we used weighted gene co-expression network (WGCNA) [8] to identify “modules” of co-expressed genes in peripheral blood of former smokers with COPD. We then used these modules to discover novel molecular pathways that are related to FEV_1_.

## Methods

### Overall study design

**Fig. 1:**
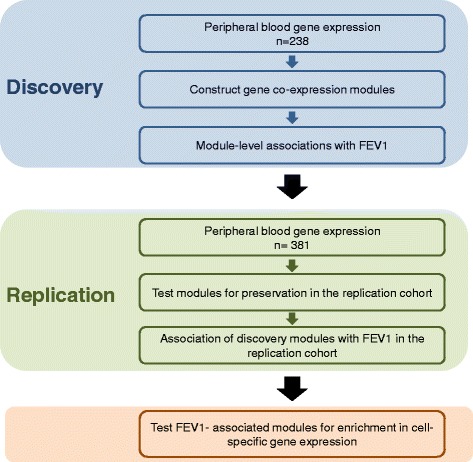
Overall study design

The overall study design is shown in Fig. 1. First, in the discovery cohort, using the WGCNA approach, we identified modules of strongly co-expressed genes. We then determined the association between these discovered gene expression modules and FEV_1_% predicted in the discovery cohort. Next, we determined the reproducibility of these relationships in an independent replication cohort. In both the discovery and replication cohorts, all analyses were performed with and without adjustment for cell counts in the peripheral circulation. We also determined whether the discovered co-expression patterns in the discovery cohort were preserved in the replication cohort. Finally, we used external cell-specific gene expression studies to determine whether the discovered gene expression modules were enriched (i.e. over-represented) for specific cell types in the peripheral circulation (e.g. neutrophils, eosinophils, lymphocytes, monocytes, etc.).

### Study subjects

The discovery and replication populations were subsets of the ECLIPSE (Evaluation of COPD Longitudinally to Identify Predictive Surrogate Endpoints) study [9]. ECLIPSE was a 3 year non-interventional, multicentre, longitudinal prospective study of COPD progression. ECLIPSE included 2164 COPD patients aged 40–75 years (smoking history ≥10 pack-years with a post-bronchodilator FEV_1_/FVC < 0.70 and FEV_1_ < 80% predicted) and 337 smokers and 245 non-smokers who were control subjects (FEV_1_/FVC > 0.70 and FEV_1_ > 90% predicted). Blood was collected in PAXgene RNA tubes and frozen at −80 °C. The gene expression sub-study of ECLIPSE was originally designed to determine gene signatures of exacerbation in peripheral blood of patients with COPD [10]. The discovery cohort consisted of 238 former smokers with COPD. The replication cohort included 381 subjects (54.3% former and 38.6% current smokers) who were not part of the discovery set. The parent ECLIPSE study was approved by the relevant ethics review boards at each of the participating centres. Study participants provided written informed consent, and participants’ information was de-identified. The ECLIPSE study was funded by GlaxoSmithKline, under ClinicalTrials.gov identifier NCT00292552 and GSK No. SCO104960. This gene expression sub-study was funded by Genome British Columbia and was approved by the Providence Health Care Research Ethics Board (REB) of the University of British Columbia (UBC) (H11-00786).

### Microarray data processing

PAXgene Blood miRNA kit from PreAnalytix (Cat. #763134) was used to extract the total RNA which was then hybridized to the Affymetrix Human Gene 1.1 ST array. Affymetrix GeneTitan MC Scanner (Affymetrix Inc.) was used to scan the array plates. The oligo Bioconductor [11] and RMA Express [12] packages were used to perform quality control on the microarray data. Background correction, normalization and summarization of the data and filtering out non-informative probe sets was undertaken using the Factor Analysis for Robust Microarray Summarization (FARMS Bioconductor package) [13]. The gene expression data are available on the NCBI Gene Expression Omnibus (GEO) under http://www.ncbi.nlm.nih.gov/geo/query/acc.cgi?acc=GSE71220.

### Weighted gene co-expression network analysis (WGCNA)

The WGCNA R package [8] was used to cluster groups of strongly co-expressed genes into co-expression networks. We followed the WGCNA tutorials at (http://www.genetics.ucla.edu/labs/horvath/CoexpressionNetwork/Rpackages/WGCNA/Tutorials/index.html). A weighted gene co-expression network reconstruction algorithm was used to create co-expression networks among the unique 18,892 genes [14]. The workflow of WGCNA began by creating a matrix of Pearson correlations between genes, and transforming these into an adjacency matrix through soft thresholding by raising it to a power β. In this study β =7 was selected so that the resulting adjacency matrix approximated a scale-free topology criterion. The adjacency matrix was transformed into a topological overlap matrix (TOM) [15]. Modules were defined as groups of highly interconnected genes. To identify modules of highly co-expressed genes, we used average linkage hierarchical clustering to group genes based on the topological overlap of their connectivity, followed by a dynamic tree-cut algorithm to cluster dendrogram branches into gene modules [16]. Each of the resulting modules was assigned a color. For each gene, we calculated a Module Membership (MM) whose values ranged between 0 and 1 by correlating the gene’s expression profile with the module eigengene determined by the first principal component of the gene expression profiles in that module. A gene that has a MM approaching 1 is considered to be highly connected to other genes in that module. In this study “hub” genes, which are considered to be central to the module, were defined based on the sum of ranks of their MM and gene significance for association with FEV_1_.

### Module preservation

To test for module preservation in the replication sample, we used the Z_summary_ statistics method of the WGCNA package [17]. The Z_summary_ is an integrated statistics of two preservation measures: a density preservation statistic which determines whether a module genes remain highly connected in the replication network, and the connectivity based preservation statistic which determines whether the connectivity pattern between genes in the discovery network is similar to that in the replication network [17]. A permutation test is used to assess the significance of the preservation statistics and Z_summary_ for the “gold” module, which is a random sample representing the entire network. Based on the thresholds determined by Langfelder et al. [17], modules with a Z_summary_ score >10 demonstrate strong preservation.

### Differential gene and module expression analysis

In the discovery dataset, linear regression was used to identify genes, whose expression was significantly related to FEV_1_ % predicted, after adjustments for age, sex and pack-years of smoking. The same approach was used to identify modules obtained from WGCNA that were differentially expressed with regards to FEV_1_% predicted. In a sensitivity analysis, both the gene and module-level associations were adjusted for cell counts in peripheral blood. For this analysis the first three principal components from the five cell types (neutrophils %, lymphocytes %, monocytes %, eosinophils % and basophils %) accounted for 99.7% of variation in the cell percentages and were used as covariates in the linear regression model. The Benjamini-Hochberg method was applied to correct for multiple testing [18].

### Replication of module FEV1 associations

For modules identified in the discovery cohort, we computed their eigenene values in the replication cohort and then tested them for associations with FEV_1_ in this independent set of subjects using linear regression. Similar to the discovery cohort the analysis was adjusted for age, sex and pack-years with additional adjustment for smoking status given that the cohort consisted of former and current smokers. A parallel analysis with additional adjustment for cell count was also performed.

### Enrichment in cell specific gene expression

The three modules associated with FEV_1_ were tested for enrichment in cell specific gene expression data from three independent studies. These include: 1) the study by Allantaz et al. [19] where they performed miRNA and mRNA expression profiling in a panel of nine human immune cell subsets (neutrophils, eosinophils, monocytes, B cells, NK cells, CD4 T cells, CD8 T cells, myeloid dendritic cells (mDCs) and plasmacytoid dendritic cells (pDCs), to identify cell-type specific expression (GSE28490 and GSE28491) in a discovery and a validation cohort, 2) the study by Naranbhai et al. [20] measured gene expression and mapped expression quantitative trait loci (eQTL) in peripheral blood CD16+ neutrophils from 101 healthy European adults (E-MTAB-3536) and 3) the study by Fairfax et al. [21] measured gene expression in B cells and monocytes (E-MTAB-945).

Affymetrix arrays were normalized using RMA and Illumina arrays were normalized using quantile normalization. Non-overlapping genes across the three studies were removed. Spearman Rank correlations were performed to determine the extent of correlation between genes in significant modules and the cell specific expression values. Furthermore, permutation was performed by shuffling the expression data for 10,000 iterations and checking the number of times that the rho is greater or equal to the value obtained for each module. In addition to *P* values, the enrichment was ranked using rho values and agreement between studies considered in the assignment of the most likely cell type.

### Ingenuity pathway analysis

QIAGEN’s Ingenuity Pathway Analysis (IPA®, QIAGEN Redwood City, www.qiagen.com/ingenuity) was used to analyze the gene sets for enriched canonical pathways.

### Statistical analysis software

All analyses were performed with R version 3.2.1 and Bioconductor packages [22]. Data processing was performed using Biovia Pipeline Pilot.

## Results

The discovery study included 238 former smokers with COPD, while the replication cohort included 323 COPD patients and 58 controls. The demographics of study participants are shown in Table 1.

**Table 1:** Subjects demographics

Variable		Discovery	Replication		*P*-value^*^
N		238 COPD	323 COPD	58 controls	
Age		64.2 ± 6.2	63.9 ± 6.1	59.6 ± 6.5	<0.001
Male		64.3%	67.8%	62.1%	0.556
BMI		28.1 ± 6	26.5 ± 5.8	28.6 ± 4.3	0.001
Smoker					<0.001
	Former	96.6%	54.5%	53.4%	
	Current	3.4%	45.5%	0%	
	Never	0%	0%	46.6%	
Pack years		46 ± 26.9	48.2 ± 26.4	26.9 ± 14.1	<0.001
FEV_1_% predicted		49.5 ± 16.2	49.7 ± 15.9	109.7 ± 15.8	<0.001
FEV_1_/FVC		0.46 ± 0.13	0.45 ± 0.11	0.79 ± 0.06	<0.001
GOLD					--
	2	43.7%	39.6%	--	
	3	44.1%	49.2%	--	
	4	12.2%	11.1%	--	
Exacerbations in prior year		1.6 ± 1.8	0.4 ± 0.5	--	<0.001

*P-value is from F test for continuous variables and chi-square test for categorical variables

### Gene level associations with FEV_1_

At the gene level, the strongest association with FEV_1_ was observed for BTN2A1 (Butyrophilin subfamily 2 member A1), which was negatively correlated with FEV_1_ (FDR = 0.094). It was the only gene that had an FDR < 0.1. The top 10 genes associated with FEV_1_ are shown in Additional file 1: Table S1.

### Module identifications and associations with FEV_1_

Applying WGCNA to the 18,892 genes expressed in blood cells led to the identification of 17 modules of various sizes ranging from 117 in the “grey60” module to 5783 genes in the “turquoise” module. A total of 3659 genes could not be mapped to any module; these genes were grouped into the “grey” module and were not considered further in the differential expression analyses. Three modules showed strong associations with FEV_1_ after adjustments for age, sex and pack-years of smoking. The most significantly correlated module was the “yellow” module containing 918 genes. It had a negative relationship with FEV_1_ (FDR = 0.004). The second strongest associated module with FEV_1_ (FDR = 0.007) was the “green” module which contained 553 genes and was also negatively correlated with FEV_1_. Finally the “brown” module which contained1569 genes was positively correlated to FEV_1_ (FDR = 0.03). The relationship between all the modules and FEV_1_ are shown in Table 2.

**Table 2:** Module associations with FEV_1_ in the discovery cohort

Module	Estimate	SE	*p*-value	FDR	Module size
Yellow	−59.924	16.106	2.49E-04	4.49E-03	918
Green	−54.373	15.998	7.96E-04	7.17E-03	553
Brown	45.964	16.373	5.42E-03	3.25E-02	1569
Greenyellow	−38.935	16.077	1.62E-02	5.97E-02	369
Blue	38.616	16.000	1.66E-02	5.97E-02	2399
Magenta	−34.945	16.104	3.10E-02	9.31E-02	455
Red	30.352	16.007	5.92E-02	1.52E-01	510
Pink	21.163	16.248	1.94E-01	4.37E-01	471
Turquoise	−19.410	16.096	2.29E-01	4.58E-01	5783
Grey60	−16.590	16.461	3.15E-01	5.66E-01	117
Midnightblue	9.907	16.225	5.42E-01	7.14E-01	152
Black	9.638	16.210	5.53E-01	7.14E-01	493
Tan	−9.510	16.190	5.58E-01	7.14E-01	330
Purple	−8.022	16.543	6.28E-01	7.14E-01	442
Lightcyan	−7.684	16.168	6.35E-01	7.14E-01	123
Salmon	−4.268	16.136	7.92E-01	8.03E-01	316
Cyan	4.047	16.209	8.03E-01	8.03E-01	233

*SE* standard error, *FDR* false discovery rate

Genes in the yellow, green and brown modules showed strong enrichment for certain biological processes, suggesting that these modules have distinct biological function (Table 3). The green module, for instance, was enriched in interleukin (IL)-10, the triggering receptor expressed on myeloid cells 1 (TREM1), the Fc Receptor-mediated phagocytosis in macrophage and monocyte and the peroxisome proliferator-activated receptors (PPAR) signalling pathways. The yellow module was enriched in IL-8 signalling, the production of nitric oxide and reactive oxygen species in macrophages, and the caveolar-mediated endocytosis and relaxin signalling pathways. The brown module was enriched in processes related to DNA transcription and translation.

**Table 3:** Biological processes enrichment for the three FEV_1_ associated modules

Canonical Pathways	*p*-value
Green Module	
IL-10 Signaling	4.47E-08
TREM1 Signaling	1.14E-06
PPAR/RXR Activation	3.96E-06
Fc Receptor-mediated Phagocytosis in Macrophages and Monocytes	9.75E-06
PPAR Signaling	9.75E-06
Yellow Module	
IL-8 Signaling	5.90E-09
Production of Nitric Oxide and Reactive Oxygen Species in Macrophages	8.84E-07
Caveolar-mediated Endocytosis Signaling	2.29E-05
Role of Tissue Factor in Cancer	2.95E-05
Relaxin Signaling	3.26E-05
Brown Module	
tRNA Charging	2.06E-10
Purine Nucleotides De Novo Biosynthesis II	5.12E-04
Cleavage and Polyadenylation of Pre-mRNA	8.27E-04
Nur77 Signaling in T Lymphocytes	1.61E-03
Leucine Degradation I	2.17E-03

### Mapping “hub” genes

The identification of modules allowed mapping of hub genes, which are central to their respective modules. To identify hub genes, we used a combination of gene significance (*P* value) for its association with FEV_1_ and the gene’s module membership (MM). MM is a measure of how well that gene is connected to the entire module and is reflective of a gene’s centrality. Using this approach, the top hub genes for the green module were dedicator of cytokinesis 5 (DOCK5) and DENN domain containing 3 (DENND3) genes. For the yellow module, the top two hub genes were RAB3D, member RAS oncogene family (RAB3D) and GRB2 (Growth factor receptor-bound protein 2) associated binding protein 2 (GAB2). For the brown module, the top hub genes were DCAF16 and EIF2AK3. The networks of GAB2, DOCK5 and DCAF16 are shown in Fig. 1.

### Impact of adjustment of complete cell count (CBC) and differential to the gene and module level associations with FEV_1_

Because peripheral blood contains a mixture of inflammatory cells, we evaluated the impact of complete cell count (CBC) and differential on gene expression at the gene as well as module level. The correlation of eigengenes with CBC in peripheral blood of the same subjects is shown in Additional file 1: Table S2 for the discovery and replication cohorts.

The yellow and green modules, which were negatively associated with FEV_1_ were positively correlated with neutrophils (*P* < 0.001) and negatively correlated to lymphocytes (*P* < 0.001) in peripheral blood. The brown module, which showed positive association with FEV_1_, was negatively correlated with neutrophils (*P* < 0.001) and positively correlated to lymphocytes (*P* < 0.001). These relationships were replicated in the replication cohort.

To evaluate the impact of peripheral blood cell count on gene level association, the analysis was repeated by including CBC and differential of peripheral blood in the statistical model we had used previously. Results are shown in Additional file 1: Table S3. The smallest FDR value after cell count adjustment was 0.64 indicating that cell count adjustment had a significant effect on peripheral blood gene expression signatures for FEV_1_. There was a modest correlation (*r* = 0.56, *P* < 2.2x10^−16^) of *P* values from the cell count and the non-cell count adjusted associations.

We performed a similar analysis by adding CBC and differential as covariates in the module level analysis (Additional file 1: Table S4). This led to the inflation of *p* values and a loss of statistical significance in the relationships between modules and FEV_1_: the yellow and green modules, for instance, ranked third and seventh with *P* values of 0.158 and 0.282, respectively and an FDR = 0.653 in the CBC adjusted analysis.

### The relationship between modules and inflammatory cells in peripheral blood

To determine which specific cell types were influencing gene expression in each of the modules, we evaluated 3 external databases that had captured cell specific gene expression in peripheral blood. The results are shown in Table 4. The green and yellow modules, which were both negatively associated with FEV_1_, were enriched in neutrophils, while the brown module, which showed positive association with FEV_1_, was enriched in CD4+ T cells, CD8+ T cells and CD56+ NK cells.

**Table 4:** Cell type enrichement for the three FEV_1_ associated modules

Reference dataset	Cell type	Module	rho*	Permutation best rho
Allantaz et al. (Discovery)	Neutrophil	Green	0.747	0.216
Allantaz et al. (Validation)	Neutrophil	Green	0.715	0.193
Naranbhai et al.	Neutrophil	Green	0.656	0.186
Allantaz et al. (Discovery)	Neutrophils	Yellow	0.773	0.147
Allantaz et al. (Validation)	Neutrophils	Yellow	0.729	0.145
Naranbhai et al.	Neutrophils	Yellow	0.697	0.143
Allantaz et al. (Discovery)	CD4+ T cells	Brown	0.618	0.121
Allantaz et al. (Discovery)	CD8+ T cells	Brown	0.589	0.127
Allantaz et al. (Validation)	CD8+ T cells	Brown	0.571	0.118
Allantaz et al. (Discovery)	CD56+ NK cells	Brown	0.568	0.122
Allantaz et al. (Validation)	CD4+ T cells	Brown	0.567	0.109
Allantaz et al. (Validation)	NK cells	Brown	0.536	0.142
Allantaz et al. (Discovery)	CD14+ monocytes	Brown	0.531	0.129

*donates *P* < 1×10^−308^ for all the reported Spearman’s rho values. Permutation best rho: the highest rho value obtained during permutation

### Modules’ preservation and reproducibility of FEV_1_ associations

**Fig. 2:**
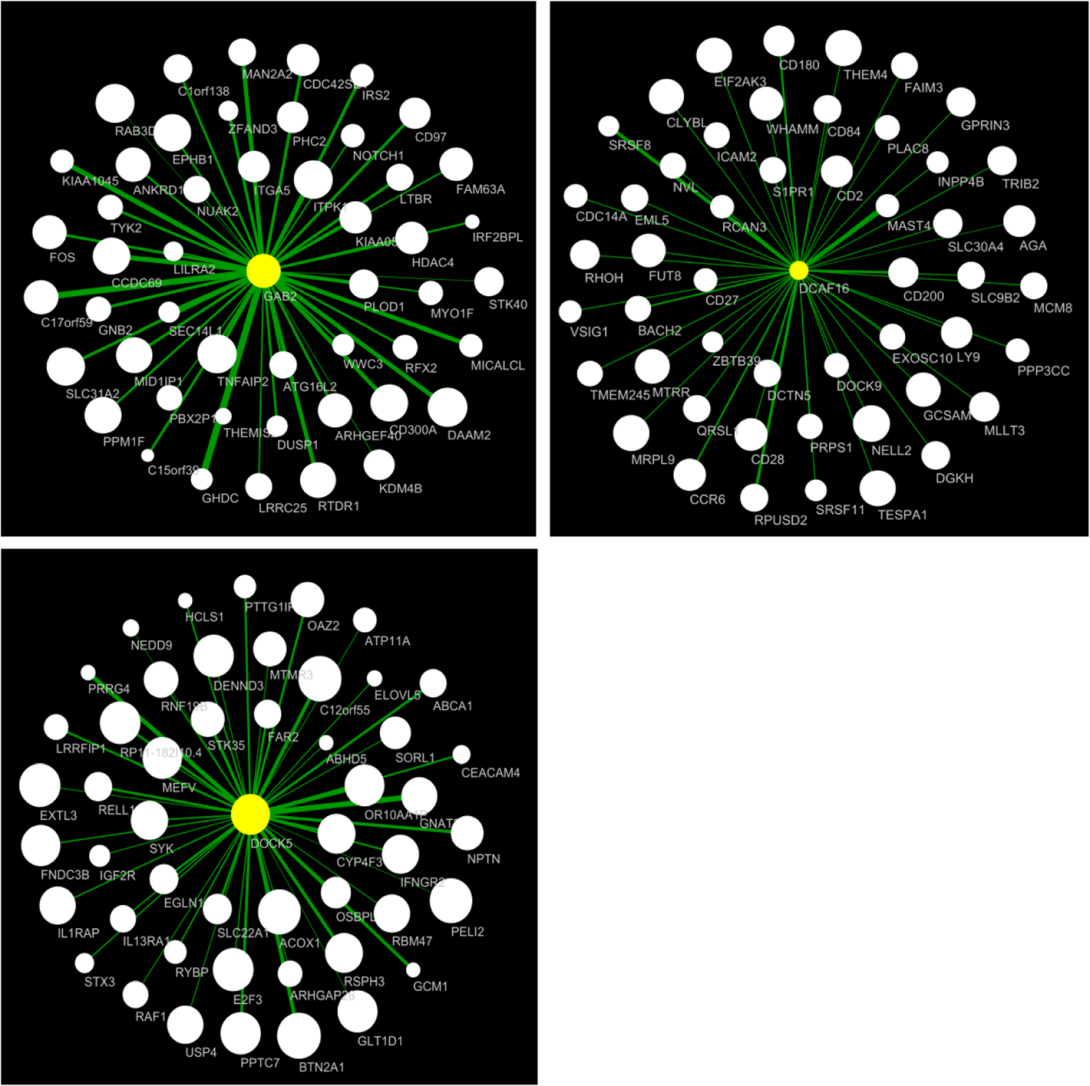
Networks of GAB2, DOCK5 and DCAF16. The figure shows the networks for GAB2, DOCK5 and DCAF16 in the *yellow*, *green* and *Brown* modules, respectively. The genes shown are top 50 significant genes that had a FDR adjusted *P* value <0.05 for association with FEV_1_. The size of the *circle* is proportional to the *P* value on the –log10 scale (larger = smaller *P* value). The thickness of the edge is proportional to the topological overlap measure (TOM) identified in WGCNA

The WGCNA modules were tested for preservation in a replication cohort of 381 current and former smokers with COPD. The resulting preservation Z_summary_ was >10, which was higher than the randomly assigned “gold” module, suggesting that all modules (except grey) were strongly preserved in the replication cohort (Fig. 2).

**Fig. 3:**
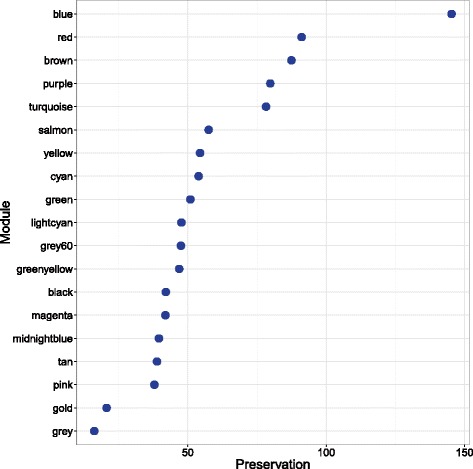
Preservation Z_summary_ of modules from discovery cohort in the replication cohort. The Y axis shows the modules vs. their corresponding Z_summary_ statistics on the X axis. All modules (except the grey modules) showed a strong preservation based on the threshold prescribed in Langfelder et al. [17] of a Z_summary_ score >10. Furthermore, the “*gold*” module consists of 1000 randomly selected genes that represent a sample of the whole genome, constructed for module preservation analysis. The *grey* module consists of genes that were not assigned to any module in the network

**Fig. 4:**
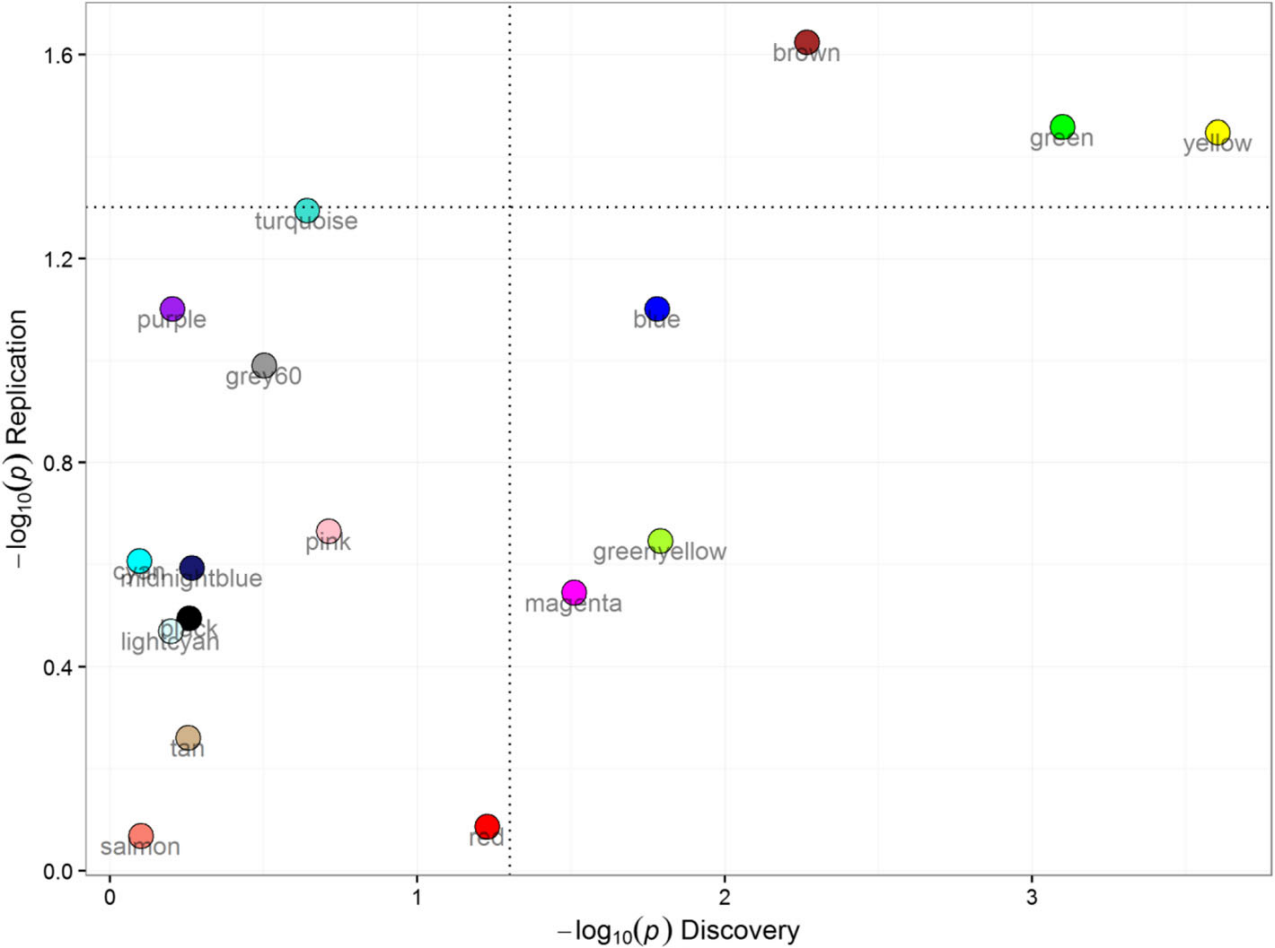
Scatter plot of module associations with FEV_1_ in discovery and replication cohorts. The Y axis shows the *P* values (−log10 scale) for FEV_1_ associations in the replication cohort while the X axis shows the association *P* values in the discovery cohort

To determine whether the module associations were reproducible, eigengenes were computed in the replication cohort for modules from the discovery cohort. The new eigengenes were then tested for association with FEV_1_ in the replication cohort (Additional file 1: Table S5). Interestingly, the top three modules associated with FEV_1_ in the discovery cohort, brown, yellow and green, were also the top three modules associated with FEV_1_ in the replication cohort with *P* = 0.024, *P* = 0.035, and *P* = 0.036 for brown, green and yellow, respectively (Figs. 3 and 4). Similar to results from the discovery cohort, adjustments for cell count in the replication cohort led to the inflation of p-values for these modules (Additional file 1: Table S6).

## Discussion

COPD is an inflammatory lung disease, which has a significant systemic component that contributes to its overall morbidity and mortality. Because inflammation is thought to play a central role in the pathogenesis of COPD, there has been a tremendous surge of interest in studying circulating immune and inflammatory cells as potential biomarkers for the disease. There is a pressing need to identify genomic signatures of disease severity and activity that can guide therapeutic decisions and address the growing burden of COPD worldwide. In this study, we used modules of co-expressed genes in a highly accessible tissue, peripheral blood, to identify genomic signatures of COPD severity using FEV_1_ as the readout.

The main findings of the present study were that: 1) At the gene level, only one gene was associated with FEV_1_ (FDR < 0.1); 2) the 18,892 genes expressed in peripheral blood mapped to 17 modules of co-expressed genes; 3) three of the modules were associated with FEV_1_, 4) in a second and larger cohort of current and former smokers with COPD and controls, all of the modules were preserved at the co-expression level, 5) the three modules in the discovery cohort that were statistically associated with FEV_1_ showed the strongest associations with FEV_1_ in the replication cohort (*P* < 0.05), 6) the two modules, which were negatively related to FEV_1_, were enriched in IL10 and IL8 pathways and were strongly correlated to neutrophil cell-specific expression, while the positively related module was enriched in DNA transcription pathways and strongly correlated to T cell specific expression.

Previous studies investigating differential expression in COPD have mainly tested genes and probesets individually; however, in vivo, genes are co-expressed in networks. By leveraging co-expression patterns, networks of closely co-expressed genes can be identified, often revealing novel functional pathways. The resulting network modules can then be tested for differential expression with FEV_1_. Another major advantage of network analyses is that this approach can significantly decrease false negatives (Type II error) by markedly reducing the number of features that are tested. In the present study the three modules reproducibly associated with FEV_1_ were enriched in biological pathways suggesting that co-expressed genes share biological functions within a particular module.

In each of the co-expressed networks, driver or “hub” genes can be identified, which can additionally inform the biology of these modules as they relate to FEV_1_. The top hub gene for the yellow module was *DOCK5* which is a member of the DOCK family of guanine-nucleotide exchange factors that activate Rho-family GTPases by exchanging bound GDP for free guanosine triphosphate (GTP) [23]. DOCK5 has been shown to interact with the regulatory and catalytic subunits of protein phosphatase 2, encoded by PPP2R1A/B/C [24]. In mice, protein phosphatase 2A has been shown to regulate innate immune and proteolytic responses to cigarette smoke exposure in the lung [25]. The top hub gene for the green module was *GAB2* which was negatively correlated to FEV_1_. GAB2 is a member of the growth factor receptor-bound protein 2 (GRB2) associated binding protein (GAB) gene family, which acts as an adapter molecule in signal transduction of cytokine and growth factor receptors, and T and B cell antigen receptors [26]. GAB2 is the principal activator of phosphatidylinositol-3 kinase in response to activation of the high affinity IgE receptor [27]. In a previous study, the expression of GAB2 in sputum was significantly increased in patients with severe emphysema compared to those who had minimal emphysema [28]. In the brown module, DDB1 and CUL4 associated factor 16 (*DCAF16*) and eukaryotic translation initiation factor 2 alpha kinase 3 (EIF2AK3) were the top two FEV_1_ hub genes. Little is known about *DCAF16*, and *EIF2AK3* encodes a protein, which functions as an endoplasmic reticulum stress sensor [29].

Although the present study is one of the largest to date that have evaluated peripheral gene expression signature in COPD [6], at the gene level, only one gene; butyrophilin subfamily 2 member A1 (BTN2A1) was significantly associated with FEV_1_. Butyrophilin has been shown to regulate immune function [30]. In contrast to gene-by-gene comparison approach, the use of network based modules identified a larger number of genes within the three significant modules which were related to FEV_1_ highlighting the value of network approaches in identifying gene signatures. Previous work on exacerbations in COPD demonstrated similar findings [31].

It is notable that adjustments for cell count had a large impact on the relationship between gene expression signatures and FEV_1_. This is not surprising given that peripheral whole blood is a heterogeneous tissue composed of many different immune cell subsets. Moreover, its cellular composition varies in response to physiological or pathological processes. These processes often involve cell differentiation and/or transit of specific cell types between blood and tissues, resulting in important shifts in the cellular makeup of samples under different conditions affecting blood-derived gene expression data. Disentangling causal from reactive relationships is challenging in observational studies. Although it is common practice to statistically adjust for peripheral blood cell composition by including CBC and differential cell counts as covariates, regression methods do not fully take into account cell-specific gene expression and thus may obfuscate important cell-specific signatures. To explore this possibility, in the present study, in addition to the standard regression analysis, we interrogated cell-specific gene expression in three external studies that contained cell-specific gene expression data that were generated by using cell isolation methods. Using this approach, we found that the two modules which were negatively associated with FEV_1_, contained strong neutrophil-specific gene expression, suggesting that increased number and/or activation of peripheral neutrophils is associated with airway obstruction. The role of neutrophils in the pathogenesis of COPD is well established [32, 33]. The module that was positively related to FEV_1_, on the other hand, contained gene expression signals that were T and B cell specific. Previous studies have highlighted the role of the adaptive immune response in COPD [34–37].

The current study has a number of limitations. First, gene expression signatures in peripheral blood may not reflect disease process in lungs of COPD patients. However, peripheral blood is more accessible than lung tissue and may provide information on biological processes such as immune responses that may be relevant in COPD. Second, FEV_1_ may not fully capture disease activity in COPD and could reflect different pathological processes (emphysema or airway disease). Finally, the cell count adjustment had a large effect on the relationship between modules and FEV_1_. Given that changes in cell abundance can be causally related to changes in FEV_1_ and disease status [38, 39] and given the strong correlations with cell specific expression in external datasets, the regression methods used for adjustment may have been overly conservative. Most published studies to date on peripheral blood in COPD do not adjust for cell count [6, 31, 40]. Future studies are warranted that incorporate differences in cell counts and/or measurement of cell specific expression changes.

## Conclusions

In conclusion, we identified gene co-expression modules in peripheral blood of patients with COPD that are highly reproducible. Three modules showed strong associations with FEV_1_ and were sensitive to cell count. In a larger replication cohort, the module-based co-expression patterns were preserved and associated with FEV_1_ in the same direction. Network based analyses represent a novel approach to discover biomarkers for COPD and warrant further attention in future studies.

## Additional file

Additional file 1: Table S1. Top 10 FEV_1_ differentially expressed genes in the discovery cohort. **Table S2**. Module correlations with cell counts in the discovery and replication cohorts. **Table S3**. Top 10 FEV_1_ differentially expressed genes in the discovery cohort after adjusting for cell count. **Table S4**. Module associations with FEV_1_ in the discovery cohort adjusting for cell count. **Table S5**. Modules association with FEV_1_ in the replication cohort. **Table S6**. Modules association with FEV_1_ in the replication cohort adjusting for cell counts. (DOCX 43 kb)

## Abbreviations

COPD: Chronic obstructive pulmonary disease
ECLIPSE: Evaluation of COPD longitudinally to identify predictive surrogate endpoints
FDR: False discovery rate
FEV_1_: Forced expiratory volume in 1 s
FEV_1_%pred: Percentage of predicted forced expiratory volume in 1 s
FEV_1_/FVC: Ratio of forced expiratory volume in 1 s to forced vital capacity
FVC: Forced vital capacity
MM: Module membership
PC: Principal component
WGCNA: Weighted gene co-expression network analysis
