# Supplementary material for: Network-based analysis reveals novel gene signatures in peripheral blood of patients with chronic obstructive pulmonary disease
Source: Respir Res. 2017 Apr 24;18:72. doi: 10.1186/s12931-017-0558-1 (PMC5404332; doi:10.1186/s12931-017-0558-1)
Supplement: Additional file 1: Table S1. — Top 10 FEV1 differentially expressed genes in the discovery cohort. Table S2. Module correlations with cell counts in the discovery and replication cohorts. Table S3. Top 10 FEV1 differentially expressed genes in the discovery cohort after adjusting for cell count. Table S4. Module associations with FEV1 in the discovery cohort adjusting for cell count. Table S5. Modules association with FEV1 in the replication cohort. Table S6. Modules association with FEV1 in the replication cohort adjusting for cell counts. (DOCX 43 kb) [file 12931_2017_558_MOESM1_ESM.docx]

**Network-Based Analysis Reveals Novel Gene Signatures of Chronic Obstructive Pulmonary Disease in Peripheral Blood**

Ma’en Obeidat^1^, Yunlong Nie^1^, Virginia Chen^2^, Casey P. Shannon^2^, Anand Kumar Andiappan^3^, Bernett Lee^3^, Olaf Rotzschke^3^, Peter J. Castaldi^4,5^, Craig P. Hersh^4,6^, Nick Fishbane^1^, Raymond T. Ng^2^, Bruce McManus^1,2^, Bruce E. Miller^7^, Stephen Rennard^8,9,^ Peter D. Paré^1,10^, Don D. Sin^1,10^

Contents

[Additional file 2](#_Toc461551953)

[Table S1. Top 10 FEV_1_ differentially expressed genes in the discovery cohort 2](#_Toc461551954)

[Table S2. Module correlations with cell counts in the discovery and replication cohorts 2](#_Toc461551955)

[Table S3. Top 10 FEV_1_ differentially expressed genes in the discovery cohort after adjusting for cell count 5](#_Toc461551956)

[Table S4. Module associations with FEV_1_ in the discovery cohort adjusting for 5](#_Toc461551957)

[cell count 5](#_Toc461551958)

[Table S5. Modules association with FEV_1_ in the replication cohort. 6](#_Toc461551959)

[Table S6. Modules association with FEV1 in the replication cohort adjusting for cell counts. 6](#_Toc461551960)

# Additional file

## Table S1. Top 10 FEV_1_ differentially expressed genes in the discovery cohort

| Gene | Estimate | SE | Statistic | P value | FDR |
| --- | --- | --- | --- | --- | --- |
| BTN2A1 | -25.961 | 5.554 | -4.675 | 5.00E-06 | 9.44E-02 |
| C16orf59 | 32.634 | 7.526 | 4.336 | 2.16E-05 | 2.04E-01 |
| C12orf55 | -11.655 | 2.801 | -4.161 | 4.46E-05 | 2.57E-01 |
| DEFB125 | 17.839 | 4.401 | 4.053 | 6.89E-05 | 2.57E-01 |
| SYNGR3 | 23.933 | 5.936 | 4.032 | 7.51E-05 | 2.57E-01 |
| DAAM2 | -7.445 | 1.859 | -4.004 | 8.38E-05 | 2.57E-01 |
| MAP1LC3B | -22.566 | 5.723 | -3.943 | 1.06E-04 | 2.57E-01 |
| RNA5SP93 | 17.458 | 4.488 | 3.890 | 1.31E-04 | 2.57E-01 |
| NDN | -21.562 | 5.587 | -3.859 | 1.48E-04 | 2.57E-01 |
| SLC31A2 | -13.922 | 3.710 | -3.753 | 2.21E-04 | 2.57E-01 |

SE: standard error. FDR: false discovery rate

## Table S2. Module correlations with cell counts in the discovery and replication cohorts

| Cell type | module | Correlation Discovery | P value  Discovery | Correlation Replication | P value  Replication |
| --- | --- | --- | --- | --- | --- |
| LYMPHP | MEbrown | 0.681 | < 1x10-308 | 0.522 | < 1x10-308 |
| LYMPHP | MEpink | 0.574 | < 1x10-308 | 0.423 | < 1x10-308 |
| NEUTP | MEgreen | 0.644 | < 1x10-308 | 0.566 | < 1x10-308 |
| NEUTP | MEgreenyellow | 0.597 | < 1x10-308 | 0.464 | < 1x10-308 |
| NEUTP | MEyellow | 0.595 | < 1x10-308 | 0.510 | < 1x10-308 |
| LYMPHP | MEgreen | -0.656 | 2.29E-30 | -0.582 | 3.58E-35 |
| NEUTP | MEbrown | -0.629 | 2.27E-27 | -0.491 | 5.04E-24 |
| LYMPHP | MEyellow | -0.624 | 6.74E-27 | -0.559 | 5.29E-32 |
| LYMPHP | MEgreenyellow | -0.598 | 2.71E-24 | -0.467 | 1.28E-21 |
| NEUTP | MEpink | -0.554 | 2.11E-20 | -0.421 | 1.90E-17 |
| LYMPHP | MEmidnightblue | 0.476 | 8.88E-15 | 0.372 | 1.15E-13 |
| NEUTP | MEmidnightblue | -0.462 | 7.06E-14 | -0.345 | 7.59E-12 |
| NEUTP | MEblack | -0.432 | 3.58E-12 | -0.330 | 6.37E-11 |
| LYMPHP | MEblack | 0.425 | 8.86E-12 | 0.326 | 1.03E-10 |
| LYMPHP | MEred | 0.357 | 1.68E-08 | 0.104 | 4.40E-02 |
| NEUTP | MEred | -0.345 | 5.26E-08 | -0.156 | 2.48E-03 |
| LYMPHP | MEblue | 0.340 | 8.67E-08 | 0.279 | 4.27E-08 |
| NEUTP | MEblue | -0.316 | 7.03E-07 | -0.269 | 1.31E-07 |
| LYMPHP | MEturquoise | -0.312 | 1.00E-06 | -0.195 | 1.51E-04 |
| NEUTP | MEsalmon | 0.306 | 1.64E-06 | 0.214 | 3.14E-05 |
| LYMPHP | MEsalmon | -0.298 | 3.13E-06 | -0.208 | 5.02E-05 |
| NEUTP | MEturquoise | 0.296 | 3.68E-06 | 0.194 | 1.61E-04 |
| FEV1PCTP | MEyellow | -0.253 | 7.81E-05 | -0.154 | 2.78E-03 |
| FEV1PCTP | MEgreen | -0.230 | 3.56E-04 | -0.151 | 3.23E-03 |
| BASOP | MEbrown | 0.209 | 1.21E-03 | -0.049 | 3.43E-01 |
| EOSP | MEblack | 0.199 | 2.08E-03 | 0.006 | 9.09E-01 |
| FEV1PCTP | MEbrown | 0.197 | 2.31E-03 | 0.162 | 1.60E-03 |
| EOSP | MEgreenyellow | -0.194 | 2.78E-03 | -0.072 | 1.66E-01 |
| EOSP | MEgreen | -0.185 | 4.35E-03 | -0.018 | 7.24E-01 |
| LYMPHP | MEgrey | -0.176 | 6.78E-03 | -0.085 | 1.01E-01 |
| BASOP | MEyellow | -0.174 | 7.55E-03 | -0.050 | 3.40E-01 |
| EOSP | MEpink | 0.172 | 8.03E-03 | 0.030 | 5.69E-01 |
| EOSP | MEbrown | 0.171 | 8.33E-03 | -0.063 | 2.26E-01 |
| FEV1PCTP | MEgreenyellow | -0.168 | 9.61E-03 | -0.129 | 1.23E-02 |
| BASOP | MEblack | 0.168 | 9.64E-03 | -0.013 | 8.08E-01 |
| EOSP | MEmidnightblue | 0.167 | 1.00E-02 | -0.162 | 1.75E-03 |
| MONOP | MElightcyan | 0.167 | 1.00E-02 | 0.080 | 1.22E-01 |
| LYMPHP | MEmagenta | -0.166 | 1.07E-02 | -0.241 | 2.47E-06 |
| EOSP | MEyellow | -0.165 | 1.09E-02 | 0.078 | 1.30E-01 |
| FEV1PCTP | MEblue | 0.161 | 1.30E-02 | 0.065 | 2.10E-01 |
| FEV1PCTP | MEmagenta | -0.155 | 1.69E-02 | -0.051 | 3.26E-01 |
| BASOP | MEgreen | -0.148 | 2.29E-02 | -0.114 | 2.71E-02 |
| NEUTP | MEgrey | 0.147 | 2.38E-02 | 0.102 | 4.82E-02 |
| MONOP | MEgreenyellow | -0.144 | 2.69E-02 | -0.044 | 3.97E-01 |
| BASOP | MEpink | 0.143 | 2.78E-02 | 0.041 | 4.27E-01 |
| EOSP | MElightcyan | 0.141 | 3.03E-02 | 0.045 | 3.85E-01 |
| MONOP | MEgreen | -0.139 | 3.22E-02 | -0.080 | 1.21E-01 |
| LYMPHP | MElightcyan | -0.135 | 3.75E-02 | -0.132 | 1.05E-02 |
| BASOP | MEgreenyellow | -0.135 | 3.83E-02 | -0.176 | 6.32E-04 |
| NEUTP | MEmagenta | 0.133 | 4.14E-02 | 0.182 | 4.04E-04 |
| FEV1PCTP | MEred | 0.125 | 5.36E-02 | 0.018 | 7.21E-01 |
| BASOP | MEturquoise | -0.123 | 5.96E-02 | 0.078 | 1.32E-01 |
| EOSP | MEsalmon | -0.119 | 6.80E-02 | -0.075 | 1.47E-01 |
| BASOP | MEred | 0.111 | 9.02E-02 | -0.024 | 6.50E-01 |
| BASOP | MEblue | 0.103 | 1.14E-01 | -0.104 | 4.58E-02 |
| EOSP | MEtan | 0.099 | 1.28E-01 | -0.068 | 1.87E-01 |
| BASOP | MEtan | 0.096 | 1.41E-01 | -0.107 | 3.88E-02 |
| NEUTP | MEpurple | 0.095 | 1.45E-01 | 0.137 | 8.07E-03 |
| MONOP | MEcyan | -0.094 | 1.48E-01 | 0.045 | 3.86E-01 |
| FEV1PCTP | MEpink | 0.093 | 1.53E-01 | 0.111 | 3.07E-02 |
| EOSP | MEturquoise | -0.093 | 1.57E-01 | 0.040 | 4.40E-01 |
| MONOP | MEred | 0.088 | 1.77E-01 | 0.156 | 2.54E-03 |
| NEUTP | MEtan | -0.088 | 1.78E-01 | -0.065 | 2.10E-01 |
| FEV1PCTP | MEturquoise | -0.084 | 1.94E-01 | -0.068 | 1.88E-01 |
| MONOP | MEsalmon | -0.084 | 2.01E-01 | 0.000 | 1.00E+00 |
| LYMPHP | MEtan | 0.081 | 2.15E-01 | 0.073 | 1.60E-01 |
| LYMPHP | MEpurple | -0.078 | 2.35E-01 | -0.130 | 1.21E-02 |
| MONOP | MEpink | 0.073 | 2.61E-01 | 0.091 | 8.05E-02 |
| BASOP | MEsalmon | -0.073 | 2.65E-01 | -0.176 | 6.61E-04 |
| MONOP | MEyellow | -0.069 | 2.94E-01 | -0.028 | 5.96E-01 |
| MONOP | MEblack | 0.068 | 2.95E-01 | 0.110 | 3.43E-02 |
| FEV1PCTP | MEgrey | -0.068 | 2.96E-01 | 0.009 | 8.56E-01 |
| MONOP | MEpurple | -0.062 | 3.45E-01 | -0.069 | 1.85E-01 |
| EOSP | MEred | 0.061 | 3.55E-01 | 0.109 | 3.57E-02 |
| EOSP | MEpurple | -0.059 | 3.66E-01 | 0.007 | 8.91E-01 |
| LYMPHP | MEcyan | 0.058 | 3.75E-01 | 0.092 | 7.69E-02 |
| EOSP | MEblue | 0.056 | 3.94E-01 | -0.059 | 2.58E-01 |
| MONOP | MEmidnightblue | 0.055 | 3.97E-01 | 0.146 | 4.71E-03 |
| BASOP | MEcyan | 0.055 | 4.03E-01 | -0.156 | 2.44E-03 |
| BASOP | MEgrey60 | 0.051 | 4.31E-01 | 0.022 | 6.65E-01 |
| LYMPHP | MEgrey60 | -0.043 | 5.08E-01 | -0.233 | 5.25E-06 |
| FEV1PCTP | MEtan | -0.042 | 5.20E-01 | 0.054 | 2.93E-01 |
| NEUTP | MElightcyan | 0.042 | 5.25E-01 | 0.096 | 6.52E-02 |
| FEV1PCTP | MEblack | 0.041 | 5.30E-01 | 0.106 | 3.95E-02 |
| MONOP | MEblue | 0.037 | 5.67E-01 | 0.106 | 3.99E-02 |
| FEV1PCTP | MEgrey60 | -0.031 | 6.39E-01 | -0.059 | 2.57E-01 |
| BASOP | MEmidnightblue | 0.031 | 6.40E-01 | -0.049 | 3.48E-01 |
| NEUTP | MEcyan | -0.030 | 6.43E-01 | -0.070 | 1.78E-01 |
| MONOP | MEtan | -0.028 | 6.65E-01 | 0.061 | 2.38E-01 |
| NEUTP | MEgrey60 | 0.028 | 6.69E-01 | 0.241 | 2.59E-06 |
| MONOP | MEmagenta | 0.027 | 6.75E-01 | 0.084 | 1.03E-01 |
| FEV1PCTP | MEsalmon | -0.024 | 7.07E-01 | -0.071 | 1.70E-01 |
| BASOP | MEpurple | 0.024 | 7.13E-01 | -0.054 | 3.02E-01 |
| FEV1PCTP | MElightcyan | -0.022 | 7.31E-01 | -0.027 | 6.04E-01 |
| FEV1PCTP | MEmidnightblue | 0.022 | 7.35E-01 | 0.067 | 1.94E-01 |
| MONOP | MEgrey60 | 0.020 | 7.58E-01 | -0.104 | 4.38E-02 |
| FEV1PCTP | MEcyan | 0.019 | 7.73E-01 | 0.050 | 3.37E-01 |
| MONOP | MEgrey | 0.018 | 7.84E-01 | -0.107 | 3.97E-02 |
| MONOP | MEturquoise | -0.016 | 8.10E-01 | -0.100 | 5.38E-02 |
| BASOP | MEgrey | -0.015 | 8.20E-01 | 0.128 | 1.35E-02 |
| EOSP | MEgrey60 | 0.013 | 8.48E-01 | 0.001 | 9.85E-01 |
| FEV1PCTP | MEpurple | -0.012 | 8.52E-01 | -0.044 | 3.96E-01 |
| EOSP | MEcyan | 0.012 | 8.59E-01 | -0.103 | 4.63E-02 |
| EOSP | MEmagenta | 0.010 | 8.73E-01 | 0.102 | 4.82E-02 |
| BASOP | MEmagenta | -0.005 | 9.43E-01 | -0.013 | 8.03E-01 |
| MONOP | MEbrown | -0.003 | 9.63E-01 | 0.088 | 8.94E-02 |
| EOSP | MEgrey | -0.003 | 9.66E-01 | -0.007 | 8.94E-01 |
| BASOP | MElightcyan | 0.003 | 9.69E-01 | -0.055 | 2.93E-01 |

## Table S3. Top 10 FEV_1_ differentially expressed genes in the discovery cohort after adjusting for cell count

| Gene | Estimate | SE | Statistic | P value | FDR |
| --- | --- | --- | --- | --- | --- |
| NDN | -22.886 | 5.411 | -4.229 | 3.39E-05 | 6.41E-01 |
| SERTAD1 | 20.513 | 5.086 | 4.033 | 7.51E-05 | 7.09E-01 |
| KLK8 | -22.644 | 5.861 | -3.864 | 1.46E-04 | 9.17E-01 |
| LOC440742 | 16.696 | 4.642 | 3.597 | 3.95E-04 | 9.92E-01 |
| GHDC | -22.603 | 6.333 | -3.569 | 4.37E-04 | 9.92E-01 |
| C4orf19 | -13.959 | 3.952 | -3.532 | 4.99E-04 | 9.92E-01 |
| DAAM2 | -6.541 | 1.872 | -3.494 | 5.71E-04 | 9.92E-01 |
| CACNB4 | -8.866 | 2.543 | -3.487 | 5.86E-04 | 9.92E-01 |
| AKR1C6P | -14.203 | 4.085 | -3.477 | 6.08E-04 | 9.92E-01 |
| DEFB125 | 14.741 | 4.285 | 3.440 | 6.91E-04 | 9.92E-01 |

SE: standard error. FDR: false discovery rate

## Table S4. Module associations with FEV_1_ in the discovery cohort adjusting for

## cell count

| Module | Estimate | SE | P value | FDR |
| --- | --- | --- | --- | --- |
| Midnightblue | -31.296 | 18.649 | 9.47E-02 | 6.53E-01 |
| Magenta | -25.621 | 15.978 | 1.10E-01 | 6.53E-01 |
| Yellow | -29.136 | 20.574 | 1.58E-01 | 6.53E-01 |
| Black | -24.49 | 17.632 | 1.66E-01 | 6.53E-01 |
| Tan | -19.01 | 15.926 | 2.34E-01 | 6.53E-01 |
| Pink | -21.129 | 19.393 | 2.77E-01 | 6.53E-01 |
| Green | -22.86 | 21.19 | 2.82E-01 | 6.53E-01 |
| Blue | 17.851 | 16.838 | 2.90E-01 | 6.53E-01 |
| Salmon | 14.224 | 16.608 | 3.93E-01 | 7.84E-01 |
| Red | 13.225 | 16.939 | 4.36E-01 | 7.84E-01 |
| Grey60 | -7.071 | 16.45 | 6.68E-01 | 9.55E-01 |
| Cyan | -3.275 | 16.026 | 8.38E-01 | 9.55E-01 |
| Greenyellow | -3.424 | 20.025 | 8.64E-01 | 9.55E-01 |
| Lightcyan | 2.352 | 16.388 | 8.86E-01 | 9.55E-01 |
| Turquoise | 2.101 | 16.531 | 8.99E-01 | 9.55E-01 |
| Purple | 1.578 | 16.359 | 9.23E-01 | 9.55E-01 |
| Brown | -1.256 | 22.033 | 9.55E-01 | 9.55E-01 |

SE: Standard error. FDR: False discovery rate

## Table S5. Modules association with FEV_1_ in the replication cohort.

| Color | Estimate | SE | P value |
| --- | --- | --- | --- |
| Brown | 52.901 | 23.287 | 2.37E-02 |
| Green | -50.127 | 23.650 | 3.48E-02 |
| Yellow | -50.093 | 23.743 | 3.56E-02 |
| Turquoise | -45.082 | 22.993 | 5.07E-02 |
| Purple | -41.776 | 23.718 | 7.91E-02 |
| Blue | 40.624 | 23.073 | 7.92E-02 |
| Grey60 | -38.235 | 23.289 | 1.02E-01 |
| Pink | 28.456 | 22.964 | 2.16E-01 |
| Greenyellow | -28.056 | 23.131 | 2.26E-01 |
| Cyan | 26.775 | 23.079 | 2.47E-01 |
| Midnightblue | 27.401 | 24.032 | 2.55E-01 |
| Magenta | -25.453 | 23.772 | 2.85E-01 |
| Black | 23.336 | 23.454 | 3.20E-01 |
| Lightcyan | 22.186 | 23.184 | 3.39E-01 |
| Tan | 14.281 | 23.830 | 5.49E-01 |
| Red | 5.116 | 22.619 | 8.21E-01 |
| Salmon | 4.172 | 22.960 | 8.56E-01 |

SE: Standard error. FDR: False discovery rate

## Table S6. Modules association with FEV1 in the replication cohort adjusting for cell counts.

| Color | Estimate | SE | P value |
| --- | --- | --- | --- |
| Turquoise | -42.970 | 23.738 | 7.12E-02 |
| Blue | 37.820 | 24.517 | 1.24E-01 |
| Purple | -34.342 | 24.174 | 1.56E-01 |
| Brown | 37.924 | 27.460 | 1.68E-01 |
| Grey60 | -31.367 | 24.432 | 2.00E-01 |
| Lightcyan | 29.265 | 23.487 | 2.14E-01 |
| Yellow | -31.711 | 28.634 | 2.69E-01 |
| Cyan | 25.551 | 23.331 | 2.74E-01 |
| Green | -30.063 | 29.073 | 3.02E-01 |
| Salmon | 22.863 | 23.878 | 3.39E-01 |
| Magenta | -18.522 | 24.892 | 4.57E-01 |
| Tan | 7.767 | 24.185 | 7.48E-01 |
| Midnightblue | 7.787 | 27.344 | 7.76E-01 |
| Pink | 7.084 | 25.472 | 7.81E-01 |
| Black | 3.066 | 25.280 | 9.04E-01 |
| Red | 1.693 | 23.191 | 9.42E-01 |
| Greenyellow | -1.691 | 26.263 | 9.49E-01 |

**SE: Standard error. FDR: False discovery rate**
